# The relevance of target product profiles for manufacturers, experiences from the World Health Organization initiative for point-of-care testing for sexually transmitted infections

**DOI:** 10.1186/s13690-021-00708-y

**Published:** 2021-10-27

**Authors:** Maurine Murtagh, Karel Blondeel, Rosanna W. Peeling, James Kiarie, Igor Toskin

**Affiliations:** 1 UNDP-UNFPA-UNICEF-WHO-World Bank Special Programme of Research, development and Research Training in Human Reproduction (HRP), World Health Organization, Geneva, Switzerland; 2 Faculty of Medicine and Health Sciences, Ghent University, Ghent, Belgium; 3 London School of Hygiene and Tropical Medicine, London, UK

**Keywords:** Sexually transmitted infections, Diagnostics, Point of care tests, Target product profiles, STI-testing, Pharmaceutical companies, Diagnostic manufacturers, Access to health, STI-strategy

## Abstract

**Background:**

Sexually transmitted infections (STIs) are a significant global public health issue that cause a high burden of disease, especially in low- and middle-income countries. Screening of key populations and early and accurate diagnosis of infection are critical. Testing for syphilis, *Chlamydia trachomatis*, *Neisseria gonorrhoeae*, *Trichomonas vaginalis*, curable STIs, as well as the human papillomavirus (HPV), is frequently unavailable in low-resource settings. Tests for these STIs that can be used at the point of patient care (POCTs) are needed.

In recent years, there has been increased attention for STI POCTs, but technical guidance, financial resources and advocacy for additional platforms/tests are required in order to foster the development of STI POCTs. The WHO Department of Sexual and Reproductive Health and Research (SRH) has developed target product profiles (TPPs), a form of technical guidance, for these STI diagnostics.

**Methods:**

SRH conducted a survey of selected companies that are developing POCTs for one or more of the STIs mentioned above to better understand how these TPPs influence the diagnostic development process – to assess their impact.

**Results:**

Survey respondents indicated that the STI POCT TPPs provided good guidance with respect to performance expectations and operational characteristics for the tests/platforms. In particular, optimal metrics for sensitivity, specificity, sample types, and time to result were considered to be very useful.

Respondents also suggested ways to improve the relevance of the STI POCT TPPs. For example, since it is often not possible for developers to achieve every desired standard, it would be useful to prioritize each performance/operational characteristic of the test and to provide a rationale as to why certain characteristics are considered important.

Respondents also emphasized the need to encourage industry participation in the TPP development process and to find creative ways, including via targeted emails, a WHO webpage directed at industry, or a coordinated communications plan to increase awareness of the TPPs.

**Conclusions:**

Companies value the STI POCT TPPs and want them to continue. In order to maximize impact, WHO should consider the proposals from the manufacturers in the interest of increasing and accelerating access to STI diagnostics and treatment in low-resource settings.

## Background

Sexually transmitted infections (STIs) continue to be a significant global public health issue, with an estimated 378 million people in 2016 becoming infected with one of 4 curable STIs: syphilis, *Chlamydia trachomatis* (CT), *Neisseria gonorrhoeae* (NG), and *Trichomonas vaginalis* (TV) [1]*.* In addition, more than 291 million women have a human papillomavirus (HPV) [1].

STIs cause a high burden of disease; they facilitate transmission of human immunodeficiency virus (HIV) and are associated with the development of certain cancers [1, 2]. Syphilis has particularly profound consequences for pregnant women, causing infant mortality as well as preterm or low weight births [3, 4]. Furthermore, CT and NG are consistently associated with tubal factor infertility and pelvic inflammatory disease [5, 6], and, together with syphilis, their sequelae may thereby contribute to subfertility and infertility [3, 5, 6].

A large proportion of STI cases are asymptomatic, confounding syndromic management of these infections using treatment algorithms based on the presence of syndromes and symptoms rather than testing. Syndromic management of STIs is often used in low- and middle-income countries (LMICs), which bear more than 90% of the global STI burden [7], but this method often fails and lacks treatment accuracy [8]. Screening key populations and early and accurate diagnosis of infection via testing are important to provide appropriate treatment, control the spread of infection and prevent adverse outcomes of STIs [9].

Where testing is available in LMICs, it typically uses technologies that require strong laboratory-based infrastructure and well-trained laboratory technicians. In addition, test turnaround time (TAT) is often long, requiring patients to return for test results on a subsequent clinic visit. However, in LMICs STI testing is often unavailable, expensive, and inaccessible. Point-of-care tests (POCTs) have shorter TATs, allowing for diagnosis and treatment in the same visit [10].

With the exception of some POCTs for the serodiagnosis of syphilis (treponemal component, single or duplex with HIV), no currently available STI POCT meets all of the so-called REASSURED criteria: none has adequate real-time connectivity and ease of specimen collection and is sufficiently affordable, sensitive, specific, user-friendly, rapid/robust, equipment free and deliverable to those who need them [11]. Currently, the test performance of CT, NG, and TV POCTs generally suffers from poor sensitivity [7]. However, sample in/answer out nucleic acid amplification tests (NAATs) offer improved performance for patient management and improved surveillance through data transmission capabilities. Such molecular tests for CT, NG, TV and HPV are already available, but too expensive for resource constrained settings. The World Health Organization (WHO) recommends the use of NAATs for screening/regular testing for asymptomatic STIs in key populations; this recommendation is conditional because the cost of NAATs is prohibitive for its availability [9].

Despite the current lack of availability of such tests, the increased attention of manufacturers on STI POCTs in recent years has resulted in a reasonably strong pipeline for such platforms suitable for use in LMICs, as evidenced by the latest landscape in STI POC diagnostics [11]. However, continued support for pipeline technologies is needed. This includes technical guidance, financial support and ongoing advocacy.

A target product profile (TPP) provides technical guidance for the development of health products, including in vitro diagnostics. WHO develops TPPs to fill the gaps of the most urgent public health needs, including concepts of access, equity and affordability as integral parts at each stage of the development process for new products [12]. In general, the WHO TPP development process consists of the following standard procedures: (i) conducting a needs assessment; (ii) appointing a TPP scientific development group; (iii) drafting an initial TPP to be revised by the development group; (iv) posting and distributing a revised draft TPP for public consultation; (v) revising and finalizing the TPP, often by convening the development group; and (vi) disseminating the final, consensus-based TPP via the WHO website and other channels.

Diagnostics are an essential component of the global priorities of the WHO. Guided by the WHO Global Strategy for the Control and Prevention of STIs, recognizing the importance of POCTs [4], the WHO Department of Sexual and Reproductive Health and Research (SRH), has developed target product profiles (TPPs) to support and accelerate the development of new STI POCTs for CT, NG, TV, syphilis and HPV, and to encourage a better fit between future STI platforms/tests and health needs in LMICs. The TPPs set out both minimal and desired performance and operational characteristics, including among other things, intended use and setting, target user, and instrument specifications. The TPPs, originally developed in 2016 are currently being updated [13].

This commentary describes the results of a survey among diagnostic manufacturers about the relevance and impact of the TPPs in their development framework as well as ways in which TPPs may be improved.

## Methods

In 2019, as part of the consensus-building process for revising the 2016 STI POCT TPPs, a brief survey was conducted among companies that manufacture or are developing one or more STI POCTs. Companies were selected for the survey from the most recent STI POCT diagnostics landscape commissioned by the WHO and available at: https://www.who.int/reproductivehealth/topics/rtis/Diagnostic-Landscape-for-STIs-2019.pdf [11, 13]. Of those approximately 25 companies, 18 companies were surveyed, including those developing/manufacturing multiplex diagnostics – i.e., those that provide, or will provide, testing for more than one infection (e.g., CT and NG) simultaneously or serially. The companies chosen are a representative sample of diagnostic companies developing or manufacturing POCTs for STIs and include both large and small diagnostic companies with POCTs for STIs already in the market as well as those with products in the pipeline.

Representatives of each of the 18 companies were contacted via email with follow-up by telephone, if required. Company representatives were generally senior scientific officers, including chief executive and chief technology officers. Representatives were asked to complete a short questionnaire indicating: 1) whether they were aware of the 2016 STI TPPs; 2) if not, what WHO could do to increase awareness; 3) if they were aware of the TPPs, whether they had consulted them; 4) if they consulted the TPPs, what they found most useful about them; and 5) how the TPPs could be improved.

Due to the low number and high variability of respondents, no statistical analysis was performed, and results are presented in a narrative.

## Results

Of the 18 companies contacted, 11 responded. The 11 respondent companies ranged from small start-ups to very large, established diagnostic companies. Most companies are developing or manufacturing NAAT-based multiplex platforms for the diagnosis of CT and NG. Some are also developing POCTs for TV and HPV. All are designed for use in LMICs.

Of the 11 companies that completed the survey, 8 were aware of the WHO-developed TPPs, and 6 of them had used them as a reference in developing their test platforms.

### Utility of TPPs

Overall, the respondents indicated that the STI TPPs provided good guidance to companies with respect to performance expectations as well as operational characteristics for the tests/platforms. The summary of requirements and characteristics found in the TPPs provided a good basis for comparison with each company’s own findings with respect to its platform, including required stability claims. In particular, optimal metrics for sensitivity, specificity, sample types, and TAT were considered to be very useful. The TPPs also provided useful guidance to companies for the recommended characteristics of tests required for use in LMICs (e.g., heat/humidity/altitude tolerances, ease of use, and cost).

### Improve relevance

Companies offered suggestions for ways to increase the relevance of and further refine the STI TPPs. Comments included that it would be useful to indicate the relative importance of each performance and operational characteristic of the test platform and to provide a rationale as to why certain characteristics are considered important in which contexts. In other words, the TPPs should prioritize the characteristics as developers indicated it is not feasible to achieve each and every desired standard. Developers often make trade-offs between factors such as speed, ease-of-use, and cost, among others. Companies also advised that TPPs should be more agnostic with respect to the technologies to be used for the platform. Rather than specifying, for example, that the test is expected to be an instrument-based molecular platform, allow that any technology and any innovative design/embodiment that meets the described characteristics in the TPP is acceptable. Companies noted the lack of attention in these TPPs to resistance testing for those infections where it is an important factor (e.g., NG). There are challenges for developers to identify viable and potentially sustainable market opportunities in LMICs. The manufacturers advised that TPPs should also link to market and funding data, to help them overcome these challenges.

### Improve industry awareness of the STI POCT TPPs

Manufacturers suggested that WHO should encourage industry participation in the TPP development process to enhance understanding of the process and the rationale for suggested product attributes. They suggested that WHO maintain and annually update a targeted email list or Listserv of multiple contacts at diagnostic companies, using this for distribution of STI POCT TPPs. Another idea was to initiate a WHO communications plan with respect to the TPPs, including publishing them in a WHO newsletter or in the WHO Bulletin, giving them visibility at professional meetings, and maintaining more direct and frequent contact at the national level in-country. More specifically, they called for the creation of a WHO webpage specifically for industry that houses TPPs and other documents relevant to diagnostic companies and developers. Finally, it was also suggested that consideration be given to developing regionalized TPPs as well as TPPs for diagnostics for high income countries.

In summary, the surveyed companies and developers value the STI POCT TPPs and want them to continue. In order to have maximum impact, there were several consistent themes: refine the TPPs, include industry more directly from the beginning of the process, and find additional ways to publicize the TPPs.

## Discussion

The WHO STI diagnostic landscapes have confirmed increased development activity for STI POCTs over the last 4–5 years, but also showed that additional platforms/tests are needed. Ongoing support for development in the form of technical guidance, financial resources and advocacy is needed. One form of technical guidance is the TPP. A brief survey with manufacturers in regard to the 2016 TPPs for STI POCTs suggested that the TPPs are useful to assist developers in designing diagnostics that are a better fit for LMICs, but in order to make them more relevant, should classify the importance of characteristics, not specify the type of technology, include characteristics for resistance and take funding and markets into account. The manufacturers also indicated ways to increase industry awareness of the TPPs, using targeted publicity and by including industry early on in the process.

The limitations of the survey include its small sample size. Only a modest number (11) diagnostic developers/manufacturers were surveyed with respect to the STI TPPs. The survey employed a simple questionnaire in order to get a snapshot of the current feedback on TPPs for STI POCTs. Risks of bias include the high number of non-respondents (7 out of 18) and the fact that companies very early in the development process for STI POC diagnostics have not been included.

Nonetheless, the survey of companies developing STI POCTs confirmed the relevance and importance of TPPs. Suggestions by companies should be taken into consideration for the WHO strategy for updating TPPs for STIs. For example, WHO could consult with industry on WHO’s proposed specifications for the TPPs (e.g., including anti-microbial resistance capabilities) and discuss with them what is most feasible for development, incorporating changes to the TPPs as deemed appropriate. WHO should intensify its advocacy efforts, broadening its communication, including both published articles and targeted online strategies.

Future research to increase access to STI POCTs in LMICs might want to explore the development and encouragement of new business models that are less concerned with maximizing profit or return on investment. Additionally, more research and tools are needed on how to introduce STI POCTs into health policies and integrate them in health systems in LMICs [7]. If more country policies would actively strive to increase STI testing for surveillance and spotting outbreaks early on, this could eventually lead to advance purchase commitments (APC), ensuring a market for STI POCTs. WHO could work with key stakeholders and opinion leaders in-country (e.g., ministries of health, regulatory authorities) to advocate and provide their support in tackling the challenges of implementation of POCTs.

## Conclusions

The 2019 WHO survey of developers and manufacturers of STI POCTs showed that manufacturers find the STI diagnostic TPPs to be useful, but that they can be improved. Industry indicated that it would like to be included earlier in the process and that TPPs could be more widely disseminated. WHO should continue to play an important role in fostering the development of POCTs for use in LMICs by regularly updating and broadly disseminating its TPPs along with its STI diagnostics landscapes, but needs to take a deeper look into how manufacturers can more meaningfully and transparently be engaged.

## Data Availability

Not applicable

## Abbreviations

CT: *Chlamydia trachomatis*
HPV: Human papillomavirus
NG: *Neisseria gonorrhoeae*
NAATs: Nucleic acid amplification tests
POCTs: Point of care diagnostic tests
STIs: Sexually transmitted infections
TPPs: Target product profiles
TV: *Trichomonas vaginalis*
TAT: Turnaround time
HRP: UNDP-UNFPA-UNICEF-WHO-World Bank Special Programme of Research, Development and Research Training in Human Reproduction
WHO: World Health Organization
SRH: WHO Department of Sexual and Reproductive Health
